# Ginsenoside Rg1 Improves PTSD‐Like Sleep Disturbances in Male Mice: Involvement of NLRP3‐Related Inflammatory and Apoptotic Pathways

**DOI:** 10.1155/np/9450146

**Published:** 2026-06-17

**Authors:** Chen Yang, Xinya Wang, Baobao Li, Shaojie Yang, Zhengrong Zhang, Jingji Wang, Xuncui Wang, Guoqi Zhu

**Affiliations:** ^1^ Key Laboratory of Xin’an Medicine, The Ministry of Education, Anhui University of Chinese Medicine, Hefei, 230038, China, ahtcm.edu.cn; ^2^ Center for Xin’an Medicine and Modernization of Traditional Chinese Medicine of IHM, and Key Laboratory of Molecular Biology (Brain Diseases), Anhui University of Chinese Medicine, Hefei, 230012, China, ahtcm.edu.cn; ^3^ The Second Affiliated Hospital of Anhui University of Chinese Medicine, Hefei, 230061, China; ^4^ Anhui Province Key Laboratory of Bioactive Natural Products, Hefei, 230012, China

**Keywords:** apoptosis, ginsenoside Rg1, NLRP3 inflammasome, posttraumatic stress disorder, sleep disturbances

## Abstract

Sleep disturbances (SD) are not merely symptoms of posttraumatic stress disorder (PTSD), but also amplify and perpetuate all other PTSD phenotypes. Ginsenoside Rg1 is extensively applied in managing neuropsychiatric diseases. Nevertheless, its impact on PTSD‐like SD and mechanisms remain largely unknown. In this study, the PTSD model in mice was produced using single‐prolonged stress (SPS) and the improvement effect of ginsenoside Rg1 on PTSD‐like behavior in mice, especially the sleep–wake phase was detected. The potential targets and signaling pathways of ginsenoside Rg1 as an intervention for treatment of PTSD with sleep disturbances (PTSD‐SD) were predicted using bioinformatics analysis. Thereafter, in vivo experiments were further used to verify the results. Our data showed that 14 days after SPS, the sleep architecture of mice was significantly disturbed, while ginsenoside Rg1 could treat PTSD‐like SD in SPS mice. The results of bioinformatics analysis suggested that NLRP‐related pathways and apoptosis may be the key factors for ginsenoside Rg1 to treat PTSD‐like sleep disturbances. Meanwhile, animal experiments also showed that the NLRP3 inflammasome, oxidative stress and apoptosis‐related markers were abnormally expressed in the hippocampus of SPS mice, but the administration of ginsenoside Rg1 could counteract the dysregulated expression of the above proteins in SPS mice. Finally, the results of docking revealed that ginsenoside Rg1 had favorable binding affinity with verified targets including NF‐*κ*B, NLRP3, ASC, GFAP, HO‐1, and cleaved caspase‐3. Collectively, ginsenoside Rg1 is effective to prevent PTSD‐like SD, likely by regulating systemic comprehensive responses.

## 1. Introduction

Posttraumatic stress disorder (PTSD) is a mental disorder caused by exposure to sudden traumatic events, which is mainly manifested by intrusive fear memories, persistent anxiety, and sleep abnormalities [1, 2]. More than 93% of individuals with PTSD experience at least one sleep phase abnormality associated with the traumatic event [3]. Lewis et al. [4] even believed that 100% of patients with war‐related PTSD would experience sleep problems. In fact, nocturnal sleep disturbances (SD) in trauma survivors play a key role in the abnormal consolidation and strengthening of trauma‐related fear emotional memories and the occurrence and development of PTSD. SD are not only common in PTSD patients but also one of the symptoms with poor clinical treatment effects. Importantly, PTSD‐like sleep disturbances demonstrate distinct clinical characteristics compared to other sleep disorders, prominently featuring insomnia, increased nightmares, and easy awakening [1, 5]. These disturbances are primarily characterized by prolonged sleep latency and marked disruption of rapid eye movement (REM) sleep architecture [6, 7]. The study also emphasized that targeted management of posttraumatic sleep symptoms can improve PTSD‐like behaviors [5, 8]. Therefore, investigating the mechanisms underlying PTSD‐like SD and exploring therapeutic interventions are of particular importance.

The hippocampus is an important brain area for learning, memory, and emotion regulation and is highly sensitive to fearful and traumatic events. In addition, the hippocampus is also a key brain region related to sleep. The hippocampus has a complex network connection with sleep‐related brain regions such as the amygdala, locus coeruleus, and hypothalamus [9]. Yang et al. [10] found that when adult rats were deprived of slow‐wave sleep or REM sleep, the neural function of the dentate gyrus of the hippocampus was inhibited, synaptic plasticity was significantly impaired, and neuronal excitability was inhibited. A PTSD‐related brain study also found that changes in sleep mediated the relationship between PTSD and hippocampal volume [11]. These data fully demonstrate that investigating brain regions implicated in both PTSD and sleep regulation, particularly the hippocampus, provides a strategic approach for elucidating the neurophysiological mechanisms underlying trauma‐associated sleep disturbances.

Traditional Chinese medicine believes that *Panax ginseng* C.A.Mey. can nourish the five internal organs, calm the spirit, stabilize the soul, stop panic, eliminate evil spirits, and calm the mind and improve intelligence. Ginsenoside Rg1 is the main active ingredient of *ginseng*, and there have been many reports on its effectiveness in treating PTSD‐like behaviors [12, 13]. Remarkably, ginsenoside Rg1 has been reported to alleviate p‐chlorophenylalanine‐induced insomnia in murine models by suppressing NOD‐, LRR‐, and pyrin domain‐containing protein 3 (NLRP3) inflammasome activation and subsequent pyroptosis but also confers neuroprotective effects through activation of the Nrf2/HO‐1 signaling pathway [14]. While sleep disturbance constitutes a hallmark symptom of PTSD, the pathogenesis of PTSD‐related sleep disturbance demonstrates substantial differences from other sleep abnormalities. However, the mechanisms underlying PTSD‐like SD and the preventive effects of ginsenoside Rg1 on these disturbances have not been reported so far.

In this study, we used the single prolonged stress (SPS) to evaluate the effects of ginsenoside Rg1 on PTSD‐like sleep disturbances and integrated network pharmacology, molecular docking, and experimental confirmation to explore its mechanisms, restoring the material basis and scientific connotation of ginseng’s calming spirit.

## 2. Materials and Methods

### 2.1. Experimental Subjects and Induction of SPS model

Two‐month‐old male C57BL/6 mice (20–25 g) were sourced from Hangzhou Ziyuan Experimental Animal Technology Co., Ltd. [Production License Number: SCXK (Zhe) 2019‐0004]. Mice were maintained under controlled conditions: a temperature of 22°C ± 2°C, a relative humidity of 55% ± 10%, and a 12 h/12 h light–dark cycle. Food and water were available *ad libitum*. The animals were euthanized by cervical dislocation following deep anesthesia induced with 2% isoflurane. The state of deep anesthesia was confirmed by the following criteria: significantly decreased respiratory rate, complete muscle relaxation, absence of eyelid reflex, and no response to toe or finger pinch. All experimental protocols adhered to ARRIVE reporting guidelines and were approved by the Ethics Committee of Anhui University of Chinese Medicine (Approval Number: AHUCM‐mouse‐2024164).

Following a week acclimation period, the SPS model was established according to previously described methodologies [15–18]. This procedure consisted of 20 min forced swimming, 2 h physical restraint, followed by ether anesthesia and subsequent electric shock (2 mA, 2 s).

### 2.2. Experimental Design and Drug Administration

#### 2.2.1. Experimental Protocol 1

**Figure 1:**
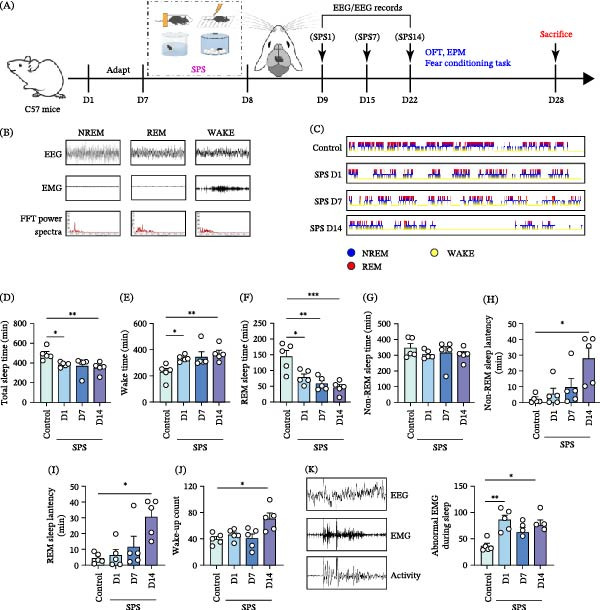
SPS induces sleep disturbances in mice. (A) Schematic of study design for Experiment 1. (B) Illustration of EEG/EMG and FFT spectra in awake, NREM, and REM sleep phases. (C) Typical EEG/EMG recordings in mice. (D–G) Time for total sleep (Kruskal–Wallis followed by Dunn’s test), WAKE (Kruskal–Wallis followed by Dunn’s test), REM (one‐way ANOVA followed by Tukey–Kramer test), and NREM (Kruskal–Wallis followed by Dunn’s test). (H) NREM sleep latency in mice (Kruskal–Wallis followed by Dunn’s test). (I) REM sleep latency in mice (Kruskal–Wallis followed by Dunn’s test). (J) Wake‐up count in mice (one‐way ANOVA followed by Tukey–Kramer test). (K) Frequency of abnormal EMG during sleep time (one‐way ANOVA followed by Tukey–Kramer test). Data are expressed as mean ± SEM (*n* = 5 per group).  ^∗^ *p* < 0.05,  ^∗∗^ *p* < 0.01, and  ^∗∗∗^ *p* < 0.001 between groups.

Following a week acclimation period, mice were randomly assigned to 4 groups (*n* = 6 per group): a control group, a SPS group (Day 1), a SPS group (Day 7), and a SPS group (Day 14) (Figure 1A).

#### 2.2.2. Experimental Protocol 2

**Figure 2:**
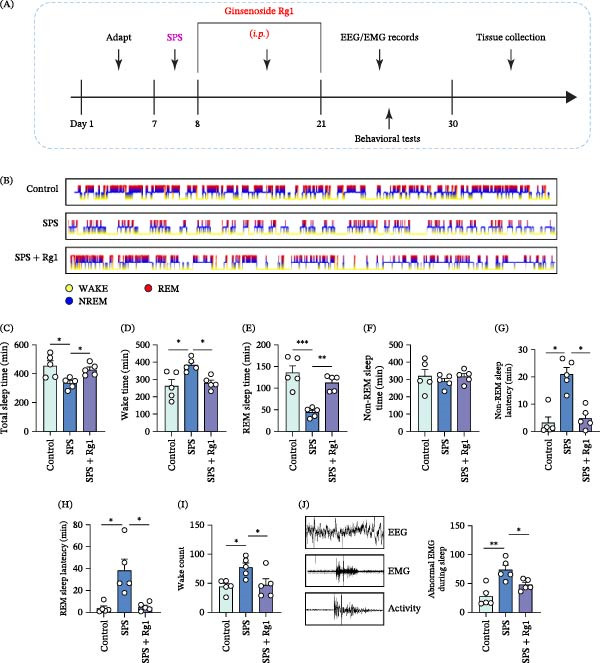
Ginsenoside Rg1 ameliorates sleep disturbances in SPS mice. (A) Schematic of study design for Experiment 2. (B) Representative EEG/EMG recordings in sleep–wake phase. (C–F) Time for total sleep, Wake, REM, and NREM in the light phase (one‐way ANOVA followed by Tukey–Kramer test). (G) NREM sleep latency in mice (Kruskal–Wallis followed by Dunn’s test.). (H) REM sleep latency in mice (Kruskal–Wallis followed by Dunn’s test.). (I) Wake‐up count in mice (one‐way ANOVA followed by Tukey–Kramer test). (J) Frequency of abnormal EMG during sleep time (one‐way ANOVA followed by Tukey–Kramer test). Data are expressed as mean ± SEM (*n* = 5 per group).  ^∗^ *p* < 0.05,  ^∗∗^ *p* < 0.01, and  ^∗∗∗^ *p* < 0.001 between groups.

After 1 week of adaptation, mice were randomly assigned to 3 groups (*n* = 8 per group): A control group, a SPS group, and a SPS + ginsenoside Rg1 (HPLC ≥98%, Shanghai, China) group. The mice in SPS + ginsenoside Rg1 group received ginsenoside Rg1 (20 mg/kg, *i.p*.) once per day for 14 days. The administration dosage of ginsenoside Rg1 was selected based on our previous report [12]. Mice in both control and SPS groups received daily administrations of equivalent volumes of saline solution for a duration of 14 consecutive days (Figure 2A).

### 2.3. EEG/EMG Recordings and Analysis

Mice were initially anesthetized with 2% isoflurane and stabilized in a stereotaxic frame. After that, the skull was exposed, and the area was cleaned with dry cotton swabs. Four holes were drilled in the skull using a craniotome: 1 mm anterior and lateral to the bregma on both sides, and 1 mm posterior and lateral to the bregma on both sides. Stainless steel screws connected to microelectrodes were fixed onto the skull and implanted to a depth of ~0.5 mm to ensure contact with the dura mater without penetration. Simultaneously, two silver wire electrodes were implanted into the trapezius muscle on both sides of the neck. After securing a micro connector with dental cement, the scalp was sutured. After that, the mice were placed in cages and used for further experiments after recovery.

The EEG/EMG recording room maintained the same environmental conditions as the housing environment: a 12 h/12 h light–dark cycle with illumination from 19:00 to 07:00, humidity at 55% ± 10%, temperature at 22°C ± 2°C, and noise levels kept below 50 dB. The microelectrodes were connected to the EEG/EMG signal acquisition system and adapted for 72 h. The Medusa software was then used to adjust the EEG channels, apply filtering, and eliminate various stimulus interferences until the EEG signals were clear and distinguishable. Recording began at 19:00, with the duration set from 19:00 to 07:00. Concurrently, a monitoring system was activated to document the mice’s activity.

According to the EEG/EMG records of mice from 19:00 to 07:00, the sleep–wake cycle of mice was divided into three phases: awakening, nonrapid eye movement (NREM) sleep, and REM sleep (Figure 1B). The sum of NREM sleep and REM sleep is the total sleep time of mice. In addition, the time from the beginning of sleep monitoring to the first transition to NREM or REM is the sleep latency, which can determine whether mice have difficulty in falling asleep. The mice in wake stage showed high EMG amplitude, low EEG amplitude, and no active activity in specific frequency band. The mice in NREM stage showed low EMG amplitude, high EEG amplitude, and 0.5–4 Hz *δ* wave activity. The mice in REM stage showed very low EMG amplitude, low EEG amplitude, and 4–8 Hz *θ* wave. To ensure objectivity and eliminate bias, all sleep assessments were conducted under strict double‐blind conditions.

### 2.4. Open‐Field Test (OFT)

Mice were adapted in the behavioral chamber for 20 min under dim lighting to facilitate free exploration. They were then gently positioned at the center of an open‐field arena (400 × 400 × 400 mm) and allowed to move freely for 5 min. The total distance moved, average velocity, time spent, and movement patterns within the central zone were recorded and analyzed using the SuperMaze behavioral analysis system (Shanghai, China).

### 2.5. Elevated Plus‐Maze (EPM)

The EPM was used to assess anxiety‐like behavior in mice. The apparatus consisted of two opposed open arms [50 (L) × 250 (W) mm], two enclosed arms [250 (L) × 50 (W) × 50 (H) mm], and a central area [50 (L) × 50 (W) mm]. Each mouse was placed on the central platform facing the enclosed arm and permitted to explore freely for 5 min. Time spent in the open arms was quantified using the SuperMaze behavioral analysis system (Shanghai, China).

### 2.6. Fear Conditioning Task (FCT)

Fear memory was assessed through a conditioning paradigm utilizing a fear conditioning chamber (UgoBasile, Italy) regulated by ANY‐Maze software (Stoelting, USA). The procedure involved one training session followed by two testing phases. During training, mice were adapted in the box with a black‐and‐white background [242 (D) × 242 (W) × 300 (H) mm] for 180 s, followed by a sound stimulus (28 s, 1 kHz, 90 dB) and a foot shock (2 s, 0.8 mA) repeated three times. After 24 h, the cued fear memory was evaluated by placing mice in the same box for 5 min without sound exposure. The freezing time was then recorded. 1 h later, the contextual fear memory was evaluated in a different environment. Following a 3 min adaptation period in a gray contextual chamber, mice were exposed to 3 min sound stimulus (4 kHz sine wave, 80 dB intensity) without foot shocks. The freezing time was recorded.

### 2.7. Target Prediction

The SMILE code of ginsenoside Rg1 was obtained using the PubChem database (https://pubchem.ncbi.nlm.nih.gov/), and then the SMILE code was imported into the Swiss Target Prediction platform (http://swisstargetprediction.ch/) to predict the target of ginsenoside Rg1. Then, the potential targets of ginsenoside Rg1 were collected based on the integrated pharmacology research platform of traditional Chinese medicine (TCMIP, http://www.tcmip.cn/) and imported into the Uniprot database (https://www.uniprot.org/) for correction of gene names. Subsequently, OMIM (https://www.omim.org/), GeneCards (www.genecards.org/), and DisGeNET (https://www.disgenet.org/) databases were used to collect PTSD‐related targets. The targets obtained in the database were merged, and duplicates were removed after screening using “relevance score*>*0.5” and “score*>*0” as conditions. Likewise, targets related to SD were obtained. The final targets of PTSD and sleep disturbances were merged as the targets of PTSD with sleep disturbances (PTSD‐SD), and the targets were imported into Uniprot database to correct the target gene name. After that, the intersection targets of ginsenoside Rg1 and PTSD‐SD were screened out as potential targets for ginsenoside Rg1 treatment of PTSD‐SD.

### 2.8. Construction of PPI Network

The common targets of ginsenoside Rg1 in treating PTSD ‐SD were submitted to the STRING database (https://cn.string-db.org/) to screen for interactions. The results were exported in TSV format and subsequently analyzed using Cytoscape software.

### 2.9. Gene Ontology (GO) and Kyoto Encyclopedia of Genes and Genomes (KEGG) Enrichment Analysis

The intersected targets were uploaded to DAVID database (https://david.ncifcrf.gov/) to analyze potential targets of ginsenoside Rg1 in treating PTSD‐SD, including GO and KEGG pathway analysis. The results of GO analysis including biological process (BP), cellular component (CC), and molecular function (MF) were sorted in descending order according to the “−log_10_ *P*” value. In addition, the results of KEGG were screened, and pathways related to neurological diseases were retained and sorted according to the “−log_10_ *P*” value. Subsequently, the top 10 results of BP, MF, and CC, along with the top 20 pathways, were imported into the SRplot online platform (https://www.bioinformatics.com.cn/) for visualization.

### 2.10. Western Blotting

Hippocampal tissues from mice were collected on ice, homogenized, and lysed. Protein concentrations were quantified using the BCA kit. Protein samples were separated by electrophoresis at 115 V for 75 min on 10% sodium dodecyl sulfate‐polyacrylamide gel, followed by transfer to a nitrocellulose membrane at 200 mA for 2 h. The membranes were then blocked with 5% nonfat milk containing 0.1% Tween‐20 at room temperature for 2 h and subsequently incubated overnight at 4°C with the following antibodies: GFAP (1:1000), IL‐1*β* (1:1000), NF‐*κ*B (1:1000), ASC (1:1000), NLRP3 (1:1000), caspase 1 (1:1000), cleaved‐caspase 3 (1:1000), and HO‐1 (1:1000). After washing three times with PBST, the membranes were incubated for 2 h at room temperature with peroxidase‐conjugated goat antirabbit or goat antimouse IgG (1:10,000). After that, membranes were developed using enhanced chemiluminescence (ECL) solution, and the scanned images were quantified using ImageJ software.

### 2.11. Measurement of Superoxide Dismutase (SOD) Activity and Malondialdehyde (MDA) Concentration

The hippocampal tissue from various groups of mice was collected on ice. SOD and MDA levels were evaluated with specialized commercial kits. Following manufacturer’s guidelines, tissue was weighed and homogenized on ice with the kit extraction buffer. After centrifugation at 12,000 rpm for 10 min, the resulting supernatants were carefully collected. Subsequently, protein concentrations was determined using the BCA kit. The absorbance values of samples were then conducted at the specified wavelengths, and the SOD activity and MDA concentration were calculated.

### 2.12. Immunofluorescence Staining

After the behavioral test, the mouse brain was dissected and immersion‐fixed in 4% paraformaldehyde for 24 h. Tissue samples were then cryoprotected by immersion in 30% sucrose solution at 4°C until complete sedimentation was achieved, followed by sectioning using a cryostat microtome. The sections were blocked with a solution containing 10% goat serum and 0.4% Triton X‐100 in PBS for 2 h at room temperature and incubated overnight at 4°C with the corresponding GFAP primary antibody (1:200) at 4°C overnight. After three thorough washes with PBS, the sections were incubated for 2 h at room temperature with FITC‐labeled antirabbit IgG and TRITC‐labeled antirabbit IgG (1:100). Nuclear counterstaining was performed by adding 4′,6‐diamidino‐2‐phenylindole (DAPI) during the final 20 min of secondary antibody incubation. Finally, the sections were mounted and imaged using an Olympus FV3000 laser scanning confocal microscope (Tokyo, Japan) and a Leica Thunder inverted microscope (Wetzlar, Germany), followed by quantitative analysis with ImageJ software.

### 2.13. Transmission Electron Microscopy (TEM)

Following anesthesia with 5% isoflurane, 1 mm^3^ hippocampal tissue samples were immediately dissected on ice and immersion‐fixed in 2.5% glutaraldehyde solution. After buffer rinsing, the tissues were fixed with 1% osmium acid for 1.5 h, followed by graded ethanol dehydration series. The processed tissues were embedded in epoxy resin, and ultrathin sectioning was performed. Sections were double‐stained with uranyl acetate and lead citrate solutions. After complete drying, ultrastructural observations of hippocampal samples were conducted using a HT7700 TEM (HITACHI, Tokyo, Japan).

### 2.14. Molecular Docking

After downloading the InChIKey code of ginsenoside Rg1 from the PubChem database, the mol2 format file of ginsenoside Rg1 was downloaded into TCMSP. Accordingly, the human pbd files of the above targets were downloaded in the RCSB PDB database (https://www.rcsb.org/). The pdb file of the receptor (protein) was preprocessed with PyMOL to remove water molecules and other atoms, and the two preprocessed files were subjected to operations such as hydrogenation, charge addition, and missing residue supplementation by AutoDock Tools software. AutoDock Vina was used for docking. Each ligand–target interaction was simulated five times to ensure the reliability of binding affinity calculations. Finally, the binding site with the lowest binding energy was selected for visualization analysis using PyMOL software.

### 2.15. Statistical Analysis

Data were expressed as mean ± standard error of the mean (SEM) and analyzed using GraphPad Prism 9.5 software. For data demonstrating normal distribution and homogeneity of variance, either Student’s *t*‐test or one‐way ANOVA followed by Tukey–Kramer post hoc test was applied. Nonnormally distributed datasets were subjected to Kruskal–Wallis nonparametric statistical testing with subsequent Dunn’s multiple comparison test. *The p* value <0.05 was considered significant.

## 3. Results

### 3.1. Sleep Disturbances are Observable in Mice Receiving SPS

The results of EEG and EMG recordings (Figure 1B,C) showed that compared with the control group, the total sleep time was significantly as early as day 1 (*p* < 0.05) and remained significantly reduced on day 14 (*p* < 0.01, *H* = 11.75, Figure 1D), and wake time was significantly increased as early as day 1 (*p* < 0.05) and remained significantly increased on day 14 (*p* < 0.01, *H* = 11.70, Figure 1E) in the SPS groups. REM sleep time was significantly reduced as early as day 1 (*p* < 0.05) and remained significantly reduced on day 7 (*p* < 0.01) and day 14 (*p* < 0.001, *F* (3, 16) = 11.33, Figure 1F). However, there was no significant difference in NREM sleep time among the groups (*p* > 0.05, *H* = 2.314, Figure 1G). In addition, both NREM sleep latency (*p* < 0.05, *H* = 10.25, Figure 1H) and REM sleep latency (*p* < 0.05, *H* = 9.466, Figure 1I) were significantly prolonged, and wake‐up count (*p* < 0.01, *F* (3, 16) = 5.549, Figure 1J) was increased significantly on day 14 after SPS regime. Notably, the number of abnormal EMG during sleep was significantly increased on days 1 (*p* < 0.01, *F* (3, 16) = 6.155, Figure 1K) and day 14 (*p* < 0.05) following SPS compared to control mice, but no significant difference was observed on day 7. Details of sleep parameters are shown in the Supporting Information (Figure S1J–N).

To ensure SPS could trigger PTSD‐like behavior, we estimated the fear memory and anxiety‐like behavior. The results demonstrated that the freezing time of mice in the SPS group was significantly increased in both contextual (*p* < 0.01, *T* (4.399) = 10, Figure S1A) and cued fear memory tests compared to the control group (*p* < 0.001, *T* (4.862) = 10, Figure S1B). In the EPM test, the SPS group showed a significant reduction in time spent in the open arms compared to the control group (*p* < 0.01, *T* (4.486) = 10, Figure S1C,H). In the OFT, the time (*p* < 0.01, *T* (3.254) = 10, Figure S1D,I) and distance (*p* < 0.001, *T* (5.601) = 10, Figure S1E, I) of the mice in the SPS group in the central area were significantly shorter than those in the control group. In addition, there was no significant difference in the mean speed (*p* > 0.05, *T* (0.5076) = 10, Figure S1F) and total distance (*p* > 0.05, *T* (0.8514) = 10, Figure S1G) traveled in the open field between the two groups, indicating that the behavior and motor functions of the mice in each group were normal.

### 3.2. Ginsenoside Rg1 Ameliorates Sleep Disturbances in SPS Mice

Our previous findings [12] and current study collectively demonstrate that ginsenoside Rg1 effectively ameliorates PTSD‐like behaviors in mice induced by SPS (Figure S2). Subsequently, we investigated the effect of ginsenoside Rg1 on the SD of SPS mice. The results of EEG and EMG recordings (Figure 2B) showed that after ginsenoside Rg1 administration, the total sleep time (*p* < 0.05, *F* (2, 12) = 6.518, Figure 2C) was significantly increased and wake time (*p* < 0.01, *F* (2, 12) = 7.276, Figure 2D) was reduced significantly. REM sleep time was significantly increased (*p* < 0.001, *F* (2, 12) = 20.36, Figure 2E). However, there was no significant difference in NREM sleep time among the groups (*p* > 0.05, *F* (2,12) = 0.3637, Figure 2F). In addition, NREM sleep latency (*p* < 0.05, *H* = 9.620, Figure 2G) and REM sleep latency (*p* < 0.05, *H* = 9.572, Figure 2H) were significantly shortened, and wake‐up count (*p* < 0.05, *F* (2, 12) = 5.320, Figure 2I) was significantly reduced. Importantly, ginsenoside Rg1 administration reduced the number of abnormal EMG during sleep in SPS mice (*p* < 0.01, *F* (2, 12) = 11.41, Figure 2J). These above results suggest that ginsenoside Rg1 improves sleep disturbances in SPS mice.

### 3.3. Target Prediction of Ginsenoside Rg1 in Rescuing PTSD‐Like SD

**Figure 3:**
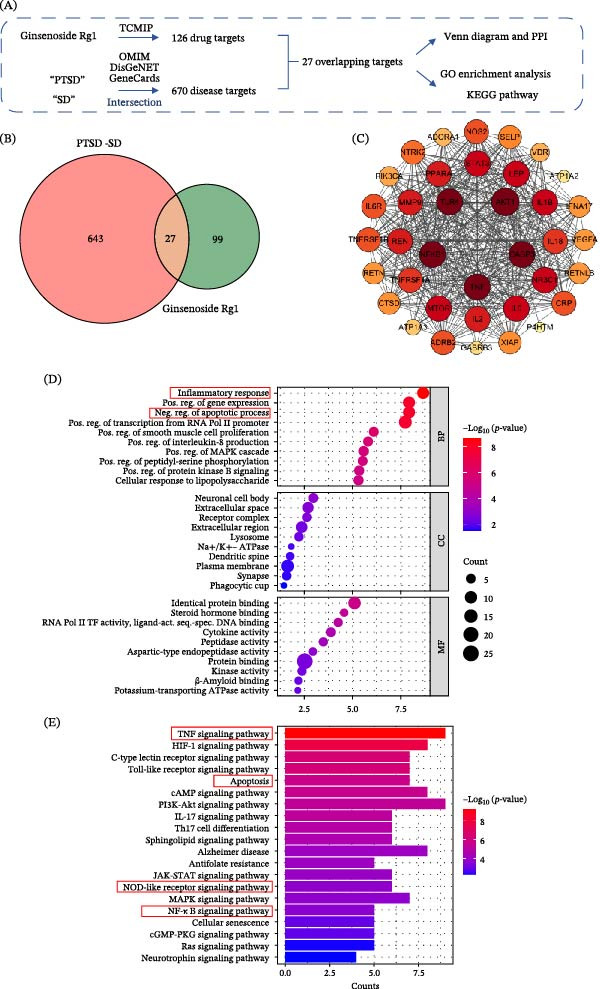
Target prediction of ginsenoside Rg1 in treating PTSD‐like sleep disturbances. (A) Schematic illustrating the bioinformatics analysis. (B) Venn diagram of PTSD‐SD and ginsenoside Rg1 targets. (C) PPI networks of common targets of PTSD‐SD and ginsenoside Rg1. (D) GO enrichment analysis. (E) Top 20 pathways of KEGG enrichment.

Later, we predicted the targets for ginsenoside Rg1 improvement in PTSD‐SD using bioinformatics analysis (Figure 3A). First, we used “Post‐traumatic stress disorder” as the search term in different databases and obtained the following results: DisGeNET screened 418 targets, OMIM screened 277 targets, and GeneCards screened 1432 targets. After removing duplicate targets, a total of 2013 PTSD‐related targets were obtained. Accordingly, we used “sleep disturbance” as the search term in different databases and obtained the following results: DisGeNET screened 525 targets, OMIM screened 275 targets, and GeneCards screened 1310 targets. After removing duplicate targets, a total of 1689 sleep disturbance‐related targets were obtained. Then, through intersection analysis of targets associated with PTSD and SD, 670 common targets were identified. Correspondingly, 126 potential targets of ginsenoside Rg1 were discerned. Finally, 27 common targets of ginsenoside Rg1 and PTSD‐SD were obtained as shown in Figure 3B.

Furthermore, the interactions of 27 common targets were drawn using the cytoscape software. The results showed that AKT, NF‐*κ*B, cleaved‐caspase 3, TNF, and interleukin‐related factors might be involved in PTSD‐like sleep disturbances (Figure 3C). The results of GO analysis showed that inflammatory response, neuronal cell bodies, and identical protein binding were involved in the BP, CC, and MF, respectively (Figure 3D). In addition, the KEGG analysis further revealed the involvement of hypoxia‐inducible factor 1 (HIF‐1) signaling, C‐type lectin receptor (CLR) signaling, Toll‐like receptor (TLR) signaling, apoptosis, cyclic adenosine monophosphate (cAMP) signaling, phosphoinositide 3‐kinase‐protein kinase B (PI3K‐Akt) signaling, and NOD‐like receptor pyrin domain‐containing (NLRP) inflammasome signaling pathway (Figure 3E).

### 3.4. SPS Induces Abnormal Protein Expression in the Mouse Hippocampus

**Figure 4:**
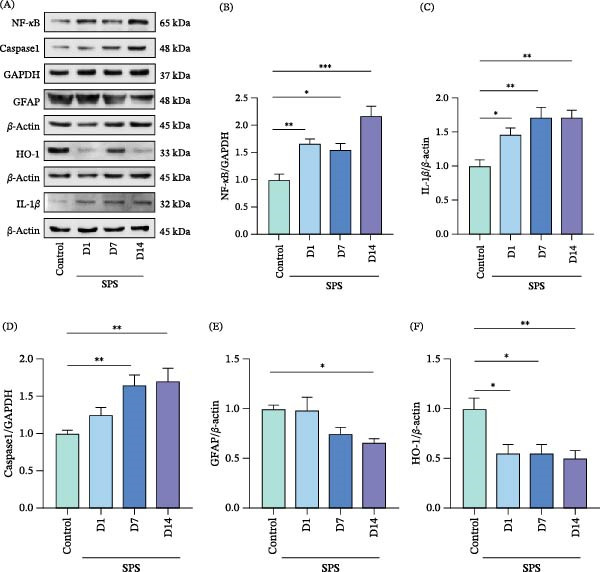
SPS induces abnormal protein expression in the hippocampus of mice. (A) Representative immunoblots of NF‐*κ*B, IL‐1*β*, Caspase 1, GFAP, and HO‐1 expression. (B) Quantitative data of NF‐*κ*B expression. (C) Quantitative data of IL‐1*β* expression. (D) Quantitative data of caspase 1 expression. (E) Quantitative data of GFAP expression. (F) Quantitative data of HO‐1 expression. Data are expressed as mean ± SEM (*n* = 5 per group).  ^∗^ *p* < 0.05,  ^∗∗^ *p* < 0.01, and  ^∗∗∗^ *p* < 0.001 between groups (one‐way ANOVA followed by Tukey–Kramer test).

In *Experimental protocol 1*, based on the results of bioinformatics analysis, we first detected the expression of inflammation, oxidative stress, and apoptosis‐related proteins in mice hippocampi on days 1, 7, and 14 after SPS stimulation (Figure 4A). The results showed that compared with the control group, the expressions of NF‐*κ*B (*p* < 0.001, *F* (3, 16) = 15.39, Figure 4B) and IL‐1*β* (*p* < 0.01, *F* (3, 16) = 9.425, Figure 4C) increased significantly in SPS groups (day 1, 7, and 14). The expression of caspase 1 increased significantly in SPS group on day 7 and 14 (*p* < 0.01, *F* (3, 16) = 7.640, Figure 4D). The expression of GFAP decreased significantly on day 14 (*p* < 0.05, *F* (3, 16) = 4.806, Figure 4E), and the expression of HO‐1 decreased significantly in SPS groups (*p* < 0.01, *F* (3, 16) = 6.639, Figure 4F). These above results suggest that SPS induces abnormal protein expression in the hippocampus, related to inflammation, oxidative stress, and apoptosis.

### 3.5. Ginsenoside Rg1 Prevents Abnormal Protein Expression and Apoptosis in the Hippocampus of SPS Mice

In *Experimental protocol 2*, to further validate the biological mechanism of ginsenoside Rg1 improving PTSD‐SD, we performed relevant animal experiments. Our results showed that compared with the control group, the protein levels of HO‐1 and glial fibrillary acidic protein (GFAP) were reduced significantly in SPS group, while the protein levels of NF‐*κ*B, NLRP3, ASC, and cleaved caspase 3 increased significantly in SPS group. The SOD activity decreased significantly, and the level of MDA increased significantly in SPS group. After ginsenoside Rg1 administration, while the protein levels of HO‐1 (*p* < 0.001, *F* (2, 12) = 70.22, Figure 5A,C) and GFAP (*p* < 0.001, *F* (2, 12) = 14.53, Figure 5A,D) increased significantly, while the protein levels of NF‐*κ*B (*p* < 0.01, *F* (2, 12) = 11.04, Figure 5A,E), NLRP3 (*p* < 0.001, *F* (2, 12) = 20.65, Figure 5B,F), ASC (*p* < 0.01, *F* (2, 12) = 13.61, Figure 5B,G), and cleaved caspase 3 (*p* < 0.001, *F* (2, 12) = 20.83, Figure 5B,H) decreased significantly in the hippocampus of SPS mice. In addition, the SOD activity (*p* < 0.05, *F* (2, 12) = 6.366, Figure 5I) increased significantly and the level of MDA (*p* < 0.05, *H* = 9.568, Figure 5J) decreased significantly in SPS mice after ginsenoside Rg1 administration. The results of immunofluorescence staining further confirmed that the positive expression of GFAP in the CA1 and DG regions of SPS mice was lower than that of the control mice, but ginsenoside Rg1 administration increased the positive cells of GFAP (*p* < 0.001, *F* (2, 12) = 16.66, Figure 5L,M; *p* < 0.001, *F* (2, 12) = 21.27, Figure 5O,P, respectively) and fluorescence intensity (*p* < 0.01, *F* (2, 12) = 11.93, Figure 5L,N; *p* < 0.01 *F* (2, 12) = 8.808, Figure 5O,Q, respectively) in the CA1 and DG regions of SPS mice. Furthermore, we observed the ultrastructure of the hippocampus by TEM. The results showed that apoptosis was observed in hippocampus of mice, which was characterized by karyopyknosis, chromatin accumulation around nucleus, and mitochondrial vacuolation. However, these changes were reversed after ginsenoside Rg1 administration (Figure 5K). These results suggest that ginsenoside Rg1 prevents abnormal protein expression and apoptosis in mice hippocampi after SPS treatment.

Figure 5Ginsenoside Rg1 prevents abnormal protein expression and apoptosis in the hippocampus of SPS mice. (A) Representative immunoblots of NF‐*κ*B, GFAP, and HO‐1 expression. (B) Representative immunoblots of ASC, NLRP3, and cleaved‐caspase 3 expression. (C–H) Quantitative data of HO‐1, GFAP, NF‐*κ*B, NLRP3, ASC, cleaved‐caspase 3 expression (one‐way ANOVA followed by Tukey–Kramer test). (I) SOD activity in hippocampus of mice (Kruskal–Wallis followed by Dunn’s test). (J) MDA concentration in hippocampus of mice (Kruskal–Wallis followed by Dunn’s test). (K) Ultrastructure of hippocampal neurons. Data are expressed as mean ± SEM (*n* = 5 per group).  ^∗^ *p* < 0.05,  ^∗∗^ *p* < 0.01, and  ^∗∗∗^ *p* < 0.001 between groups. (L) Representative immunofluorescence of GFAP expression in the CA1 region of mouse hippocampus. (M) Positive cells of GFAP in the CA1 region of mouse hippocampus. (N) Fluorescence intensity of GFAP expression in the CA1 region of mouse hippocampus. (O) Representative immunofluorescence of GFAP expression in the DG region of mouse hippocampus. (P) Positive cells of GFAP in the DG region of mouse hippocampus. (Q) Fluorescence intensity of GFAP expression in the DG region of mouse hippocampus. Data are expressed as mean ± SEM (*n* = 5 per group).  ^∗^ *p* < 0.05,  ^∗∗^ *p* < 0.01, and  ^∗∗∗^ *p* < 0.001 between groups (one‐way ANOVA followed by Tukey–Kramer test).

**Figure:**
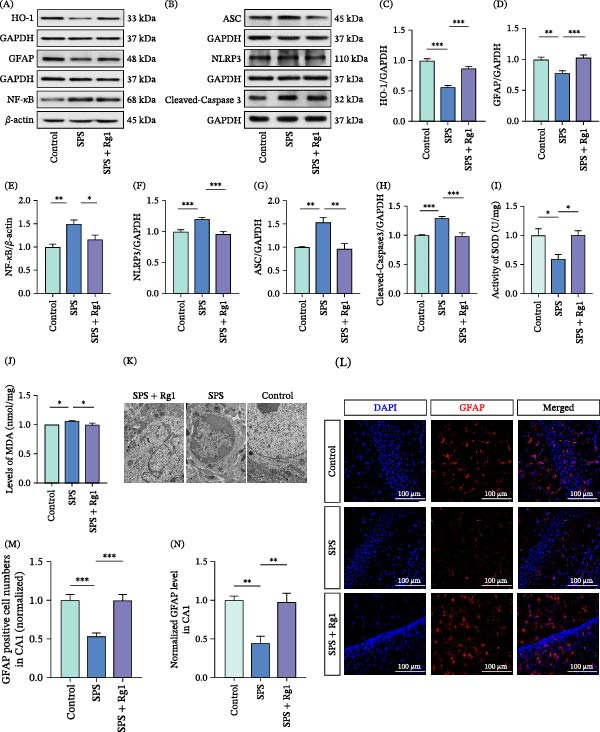

**Figure:**
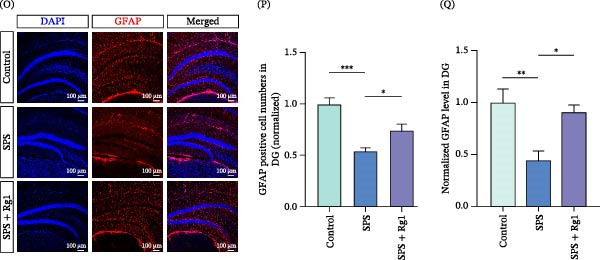

### 3.6. Docking Analysis of Ginsenoside Rg1 and Verified Targets

**Figure 6:**
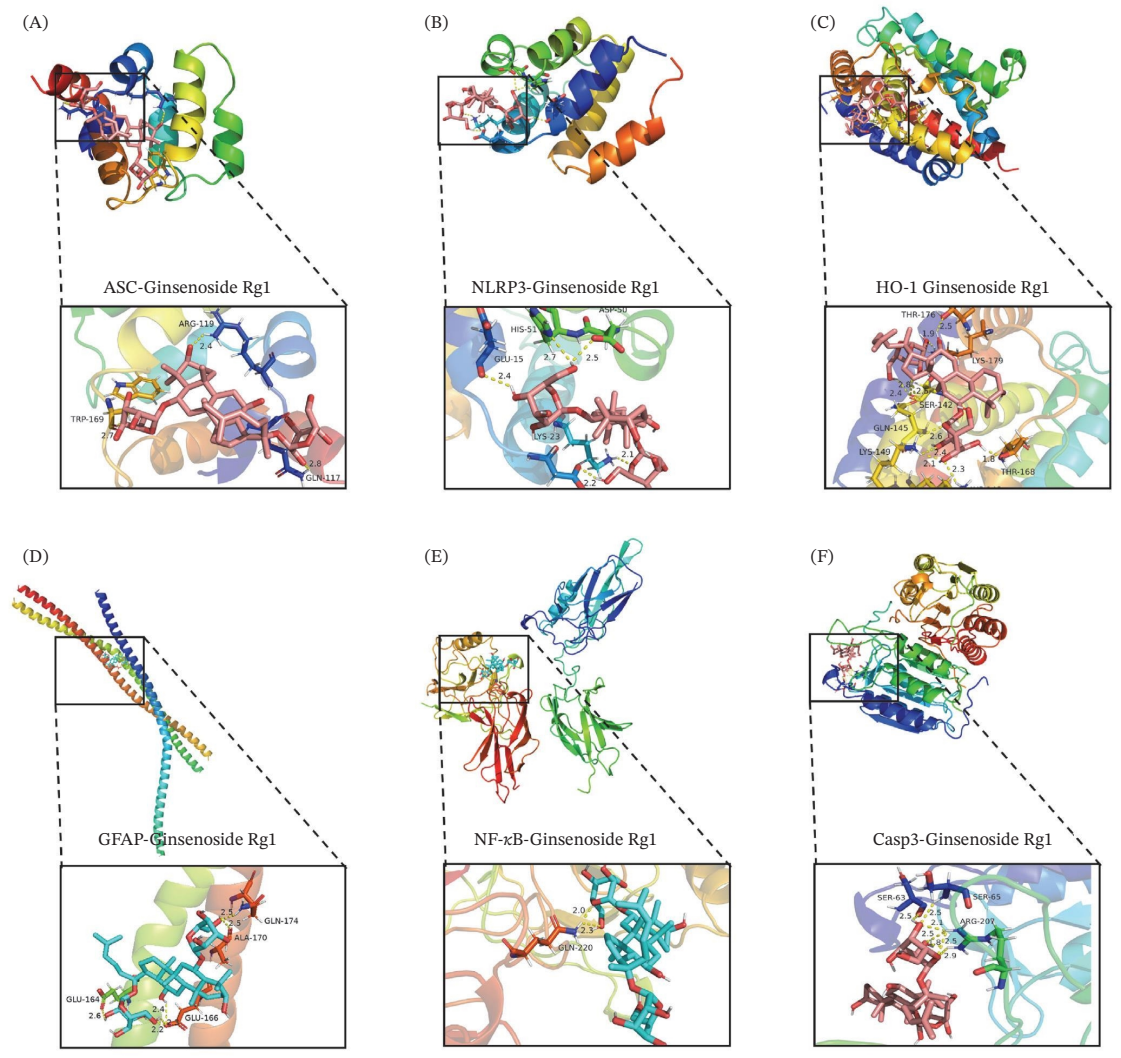
Schematic diagram of docking between ginsenoside Rg1 and verified targets. (A) Docking of ginsenoside Rg1 and ASC. (B) Docking of ginsenoside Rg1 and NLRP3. (C) Docking of ginsenoside Rg1 and HO‐1. (D) Docking of ginsenoside Rg1 and GFAP. (E) Docking of ginsenoside Rg1 and NF‐*κ*B. (F) Docking of ginsenoside Rg1 and cleaved‐caspase 3.

To evaluate the interaction between ginsenoside Rg1 and the verified targets, docking of ginsenoside Rg1 with NF‐*κ*B, NLRP3, ASC, GFAP, HO‐1, and Caspase‐3 was performed (Table 1). The binding energy score between ligands and receptors is commonly employed as one of the criteria for evaluating molecular interactions. A binding energy value below −5.0 kcal/mol indicates favorable binding activity, while a binding energy value below −9.0 kcal/mol demonstrates strong binding affinity [19]. Among all the docking results, the site with the lowest binding energy in each group was selected and visualized using PyMOL software. The results showed that the arginine ARG‐119, glutamine GLN‐117, and tryptophan TRP‐169 residues of ginsenoside Rg1 formed stable hydrogen bonds with ASC (Figure 6A). The aspartic acid ASP‐50 and ASP‐19, histidine HIS‐51, glutamic acid GLU‐15, and lysine LYS‐23 residues of ginsenoside Rg1 form stable hydrogen bonds with NLRP3 (Figure 6B). Threonine THR‐176 and THR‐168, lysine LYS‐179, LYS‐149, and LYS‐148, serine SER‐142, and glutamine GLN‐145 residues of ginsenoside Rg1 form stable hydrogen bonds with HO‐1 (Figure 6C). The glutamine GLN‐174, glutamic acid GLU‐166, and phenylalanine ALA‐170 residues of ginsenoside Rg1 form stable hydrogen bonds with GFAP (Figure 6D). The glutamine GLN‐220 residue of ginsenoside Rg1 forms a stable hydrogen bond with NF‐*κ*B (Figure 6E). Serine SER‐63, Serine SER‐65, and Arginine ARG‐207 residues of ginsenoside Rg1 form stable hydrogen bonds with cleaved caspase 3 (Figure 6F).

**Table 1:** Docking analysis of ginsenoside Rg1 and verified targets.

Molecule name	Affinity (kcal/mol)	Affinity (kcal/mol)	Affinity (kcal/mol)	Affinity (kcal/mol)	Affinity (kcal/mol)	Affinity (kcal/mol) $\bar{\chi }\pm S$
ASC	−5.2	−5.0	−5.6	−7.3	−7.6	−5.27 ± 0.306
NLRP3	−6.3	−6.2	−6.3	−8.5	−8.2	−6.27 ± 0.058
GFAP	−5.5	−6.3	−6.0	−6.4	−7.1	−5.93 ± 0.404
NF‐*κ*B	−8.1	−8.3	−8.2	−6.5	−7.4	−8.2 ± 0.1
Caspase3	−8.2	−8.0	−7.9	−7.7	−7.3	−8.03 ± 0.153
HO‐1	−6.5	−6.7	−6.8	−7.6	−8.0	−6.67 ± 0.152

## 4. Discussion

In this study, we demonstrated that ginsenoside Rg1 improved the sleep‐awake phase and sleep quality of SPS mice, promoted the expression of antioxidant stress proteins, and reduced the inflammatory response and apoptosis in the hippocampus of mice. These results suggest that ginsenoside Rg1 may improve PTSD‐like behaviors with sleep disturbances by regulating systemic comprehensive responses.

Consistent with our previous report [12], we found that SPS mice showed anxiety‐like behavior and abnormal fear memory. It is worth noting that SPS mice also showed SD, which were manifested by shortened total sleep amount, increased awakening time and frequency, and prolonged sleep latencies, especially a significant reduction in REM sleep time. REM sleep is known to help erase fear memories, and the disruption of REM sleep after a traumatic event could increase the risk of PTSD [20, 21]. Surprisingly, our findings revealed that SPS induced a significant increase in the number of abnormal EMG during sleep in mice, indicating that abnormal EMG activity may affect the stability of sleep, especially REM sleep [22]. These findings suggest that PTSD‐like sleep disturbances, especially REM sleep, may be key factors promoting the occurrence of PTSD. It has been reported that ginsenoside Rg1 administration significantly increased the total sleep time [23], the duration of REM and NREM sleep, the number of NREM sleep occurrences, and reduced the duration of awake episodes in rats [24]. Consistently, our results showed that ginsenoside Rg1 could significantly increase the total sleep time, especially REM sleep time, and reduce the wake time and count, as well as the sleep latency of NREM and REM sleep in SPS mice. Importantly, ginsenoside Rg1 could significantly reduce abnormal EMG during sleep in SPS mice. These findings suggest that ginsenoside Rg1 improves SD and sleep quality in SPS mice, which may be the primary prerequisite for preventing the occurrence of PTSD symptoms.

Individuals with PTSD exhibit significantly elevated levels of proinflammatory markers [25]. We and others also confirmed that IL‐6, IL‐1*β* and TNF‐*α* increased in the prefrontal cortex and hippocampus of SPS‐induced mice [18, 26]. In addition, the increase of inflammatory response may also be caused by sleep disturbances, which may be an important mechanism linking PTSD and sleep [27]. Notably, PTSD has recently been found to be associated with activation of NLRP3 inflammasome in astrocytes of GFAP‐GFP transgenic mice [28]. Consistent with this report, we previously reported that Anshen Dingzhi prescription, which mainly contains *ginseng*, can target the hippocampal deleted in colorectal cancer (DCC) to regulate the expression of inflammatory proteins NLRP3, NF‐*κ*B and IL‐6 in SPS mice [29]. Interestingly, a recent study has shown that REM sleep deprivation can induce anxiety and depression‐like behavior in mice, and the levels of NF‐*κ*B and NLRP3 inflammasome including NLRP3, ASC, and caspase‐1 are significantly increased in the hippocampus [30]. In fact, there are no reports on network pharmacology analysis related to posttraumatic SD. In this present study, we found that the cAMP signaling pathway, PI3K/Akt, TNF, NGF, and apoptosis were involved in ginsenoside Rg1 treatment of PTSD with sleep disturbances using bioinformatics analysis. Furthermore, our results of animal experiments showed that NLRP3 inflammasome‐related protein expression was significantly increased in the hippocampus of SPS mice characterized by significantly reduced REM sleep, while ginsenoside Rg1 administration reversed the abnormal expression of inflammatory proteins in SPS mice. In addition, changes in GFAP expression may reflect inflammatory states associated with PTSD [31]. Our data showed that ginsenoside Rg1 could inhibit the decreased GFAP expression in SPS mice. Although ginsenoside Rg1 is predominantly water soluble and exhibits poor intestinal permeability and low bioavailability, its ability to access the brain, especially hippocampal glial cells, may be explained by several nonexclusive mechanisms. First, Rg1 could be transported across cellular membranes via organic anion‐transporting polypeptides (OATPs) or other carrier‐mediated processes, which have been implicated in the uptake of various ginsenosides. Second, in the central nervous system, blood‐brain barrier permeability may be transiently increased under pathological conditions, facilitating parenchymal distribution. Third, internalization into glial cells might occur through endocytic pathways, followed by endosomal escape into the cytoplasm. Additionally, despite low oral bioavailability, systemic exposure after parenteral administration or accumulation through repeated dosing could still yield sufficient concentrations in the brain interstitial fluid to permit subsequent cellular uptake. Further studies investigating the precise uptake mechanisms and subcellular trafficking of ginsenoside Rg1 in glial cells would help clarify how it reaches the intracellular targets identified in this study.

Oxidative stress is caused by an imbalance between the ROS production and antioxidant defense systems. Mitochondrial ROS can mediate apoptosis through cytochrome C and activation of caspase [32]. Studies have shown that preventing oxidative stress is effective to improve PTSD‐like behavior [33, 34]. Furthermore, in rodent models, stress causes atrophy or death of prefrontal cortex, hippocampal neurons, and glial cells [35–37]. Indeed, in the sleep deprivation model, elevated levels of proinflammatory markers were found to contribute to cardiac damage and metabolic disorders by promoting oxidative stress, primarily through upregulation of iNOS/NO/NF‐*κ*B/caspase3 signaling [38]. Therefore, oxidative stress‐mediated apoptosis may be the common pathological mechanism of PTSD and SD. A previous study has demonstrated that ginsenoside Rg1 mitigates stress‐induced depression‐like behaviors by reducing neuronal oxidative stress and inflammatory responses [39]. Meanwhile, ginsenoside Rg1 has been shown to activate the BDNF signaling pathway and promote neurogenesis [39]. Based on these established mechanisms and bioinformatic predictions, we selected oxidative stress‐ and apoptosis‐related markers to evaluate the therapeutic potential of ginsenoside Rg1 on PTSD‐like behavior with sleep disturbances. The results demonstrated that SPS mice exhibited significantly reduced expression of antioxidant markers, elevated peroxidation levels, and evident hippocampal apoptosis, while ginsenoside Rg1 could significantly prevent the occurrence of oxidative stress and apoptosis in SPS mice. Furthermore, the results of docking showed that ginsenoside Rg1 exhibited favorable binding activity with HO‐1 and caspase‐3, suggesting that ginsenoside Rg1 may exert its effect by targeting these key molecules.

Sleep disturbance is a key initiating factor for PTSD symptoms and poor prognosis. Future studies will focus on exploring the early manifestations and hallmarks of PTSD symptoms, and timely targeted interventions should be carried out as soon as possible. Ginsenoside Rg1 may exert its effects by regulating the overall systemic response and mobilizing the body’s self‐repair ability. It has been reported that Rg1 is a potential ligand for glucocorticoid receptors [40], and activation of glucocorticoid receptors is involved in the control of inflammatory responses [41]. Our research group has previously reported that the inflammatory response is an important pathology of PTSD [18]. Therefore, ginsenoside Rg1 may also exert its intervention in PTSD through other mechanisms.

However, this study has not yet clarified how ginsenoside Rg1 prevents the occurrence of PTSD symptoms by improving trauma‐induced sleep abnormalities. Although our findings indicate that ginsenoside Rg1 may ameliorate PTSD‐like SD by attenuating hippocampal inflammation, oxidative stress, and apoptosis, the current study lacks pharmacological interventions and genetic approaches to definitively identify its specific molecular targets and clarify the underlying mechanisms. Furthermore, due to the inherent uncertainties of bioinformatics simulations, the results of this study only suggest the potential binding between ginsenoside Rg1 and the aforementioned targets. Future studies will employ more direct molecular interaction techniques for validation. Our previous study has demonstrated that administration of ginsenoside Rg1 alone in control animals had no significant differences compared with the control group in behavioral tests. This suggests that ginsenoside Rg1 may not have any effect on normal animals, and its effects are primarily manifested as an intervention in the pathological state induced by SPS [12].

## 5. Conclusion

**Figure 7:**
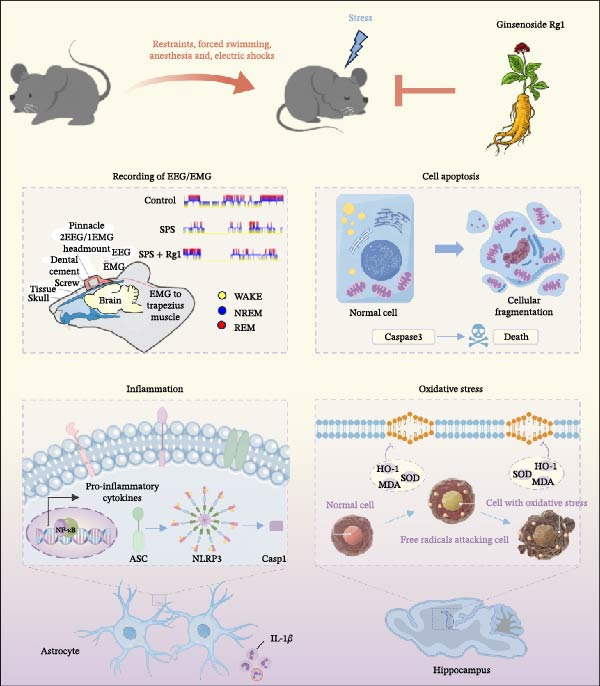
Schematic illustration of the potential mechanisms. Ginsenoside Rg1 improves PTSD‐like behavior with sleep disturbances by preventing the activation of NLRP3 inflammasome, oxidative stress, and apoptosis.

Our data demonstrate that ginsenoside Rg1 effectively prevents PTSD‐like sleep disturbances. The inhibition of NLRP3 inflammasome activation, oxidative stress, and apoptosis may underlie the protective effects of ginsenoside Rg1 (Figure 7). These findings will provide experimental basis for ginsenoside Rg1 to intervene in PTSD‐like SD.

## Author Contributions

**Chen Yang:** conceptualization, data curation, writing – original draft preparation. **Xinya Wang:** conceptualization, data curation, project administration, funding acquisition, writing – review and editing. **Baobao Li and Zhengrong Zhang:** methodology. **Shaojie Yang:** validation. **Jingji Wang:** resources. **Guoqi Zhu:** project administration, funding acquisition, writing – review and editing.

## Funding

This research was funded by the Anhui Natural Science Foundation (Grant 2208085MH282), the Key Project of Anhui Natural Science Research (Grants 2024AH051045 and 2024AH050946), Research Funds of Center for Xin’an Medicine and Modernization of Traditional Chinese Medicine of IHM (Grants 2023CXMMTCM013 and 2023CXMMTCM021), and Basic‐Clinical Integration Program of Anhui University of Chinese Medicine (Grant JCLCA2025003).

## Disclosure

All authors have read and agreed to the published version of the manuscript.

## Ethics Statement

The animal study protocol was approved by the Experimental Animal Ethics Committee of Anhui University of Chinese Medicine (Hefei, Anhui, China) (protocol code: AHUCM‐mouse‐2024164 and date of approval: October 10, 2024).

## Conflicts of Interest

The authors declare no conflicts of interest.

## Supporting Information

Additional supporting information can be found online in the Supporting Information section.

## Supporting information


**Supporting Information** Figure S1: SPS induces PTSD‐like behavior and sleep disturbances in mice. (A) Freezing time of contextual fear memory (Independent student *t*‐test). (B) Freezing time of cued fear memory (Independent student *t*‐test). (C) Time spent exploring the open arm in the elevated plus‐maze test (Independent student *t*‐test). (D) Time spent in the center area in the open‐field test (Independent student *t*‐test). (E) Distance traveled in the central area in the open‐field test (Independent student *t*‐test). (F) Total distance in the open‐field test (Independent student *t*‐test). (G) Mean speed in the open‐field test (Independent student *t*‐test). (H) Representative traces in the elevated plus‐maze test (Independent student *t*‐test). (I) Representative traces in the open‐field test (Independent student *t*‐test). (J–M) Hourly REM, NREM, Sleep, WAKE time duration in mice during the 19:00–7:00 monitoring time range (Two‐way ANOVA). (N) Pie chart of sleep chronology in mice. Data are expressed as mean ± SEM.  ^∗^ *p* < 0.05,  ^∗∗^ *p* < 0.01, and  ^∗∗∗^ *p* < 0.001 between groups. Figure S2: Ginsenoside Rg1 prevented PTSD‐like behavior and SD in SPS mice. (A) Freezing time of contextual fear memory (one‐way ANOVA followed by Tukey–Kramer test). (B) Freezing time of cued fear memory (one‐way ANOVA followed by Tukey–Kramer test). (C) Time spent exploring the open arm in the elevated plus‐maze test (one‐way ANOVA followed by Tukey–Kramer test). (D) Distance traveled in the central area in the open‐field test (one‐way ANOVA followed by Tukey–Kramer test). (E) Time spent in the center area in the open‐field test (one‐way ANOVA followed by Tukey–Kramer test). (F) Mean speed in the open‐field test (one‐way ANOVA followed by Tukey–Kramer test). (G) Total distance in the open‐field test (one‐way ANOVA followed by Tukey–Kramer test). (H) Representative traces in the open‐field test (one‐way ANOVA followed by Tukey–Kramer test). (I) Representative traces in the elevated plus‐maze test (one‐way ANOVA followed by Tukey–Kramer test). (J–M) Hourly REM, NREM, Sleep, WAKE time duration in mice during the 19:00–7:00 monitoring time range (Two‐way ANOVA). (N) Pie chart of sleep chronology in mice. Data are expressed as mean ± SEM.  ^∗^ *p* < 0.05,  ^∗∗^ *p* < 0.01, and  ^∗∗∗^ *p* < 0.001 between groups.

## Data Availability

All the data are contained within the article, and the data presented in this study are available upon request from the corresponding author.
