# Supplementary material for: Ginsenoside Rg1 Improves PTSD‐Like Sleep Disturbances in Male Mice: Involvement of NLRP3‐Related Inflammatory and Apoptotic Pathways
Source: Neural Plast. 2026 Jun 17;2026:9450146. doi: 10.1155/np/9450146 (PMC13273226; doi:10.1155/np/9450146)
Supplement: Supplementary file 1 — Supporting Information Figure S1: SPS induces PTSD‐like behavior and sleep disturbances in mice. (A) Freezing time of contextual fear memory (Independent student t‐test). (B) Freezing time of cued fear memory (Independent student t‐test). (C) Time spent exploring the open arm in the elevated plus‐maze test (Independent student t‐test). (D) Time spent in the center area in the open‐field test (Independent student t‐test). (E) Distance traveled in the central area in the open‐field test (Independent student t‐test). (F) Total distance in the open‐field test (Independent student t‐test). (G) Mean speed in the open‐field test (Independent student t‐test). (H) Representative traces in the elevated plus‐maze test (Independent student t‐test). (I) Representative traces in the open‐field test (Independent student t‐test). (J–M) Hourly REM, NREM, Sleep, WAKE time duration in mice during the 19:00–7:00 monitoring time range (Two‐way ANOVA). (N) Pie chart of sleep chronology in mice. Data are expressed as mean ± SEM. ∗ p < 0.05, ∗∗ p < 0.01, and ∗∗∗ p < 0.001 between groups. Figure S2: Ginsenoside Rg1 prevented PTSD‐like behavior and SD in SPS mice. (A) Freezing time of contextual fear memory (one‐way ANOVA followed by Tukey–Kramer test). (B) Freezing time of cued fear memory (one‐way ANOVA followed by Tukey–Kramer test). (C) Time spent exploring the open arm in the elevated plus‐maze test (one‐way ANOVA followed by Tukey–Kramer test). (D) Distance traveled in the central area in the open‐field test (one‐way ANOVA followed by Tukey–Kramer test). (E) Time spent in the center area in the open‐field test (one‐way ANOVA followed by Tukey–Kramer test). (F) Mean speed in the open‐field test (one‐way ANOVA followed by Tukey–Kramer test). (G) Total distance in the open‐field test (one‐way ANOVA followed by Tukey–Kramer test). (H) Representative traces in the open‐field test (one‐way ANOVA followed by Tukey–Kramer test). (I) Representative traces in the elevated plus‐maze test (one‐way [file NP-2026-9450146-s001.docx]

**Ginsenoside Rg1 improves PTSD-like sleep disturbances in male mice: Involvement of NLRP3-related inflammatory and apoptotic pathways**

Chen Yang^1,2^, Xinya Wang^1,2^, Baobao Li^1,2^, Shaojie Yang ^2,3^, Zhengrong Zhang^1,2^, Jingji wang ^2,3^, Xuncui Wang^1,2,4,*^, Guoqi Zhu^1,2,*^

^1^ Key Laboratory of Xin’an Medicine, the Ministry of Education, Anhui University of Chinese Medicine, Hefei 230038, China

^2^ Center for Xin’an Medicine and Modernization of Traditional Chinese Medicine of IHM, and Key Laboratory of Molecular Biology (Brain diseases), Anhui University of Chinese Medicine, Hefei 230012, China

^3^ The Second Affiliated Hospital of Anhui University of Chinese Medicine, Hefei 230061, China

^4^ Anhui Province Key Laboratory of Bioactive Natural Products, Hefei 230012, China

**
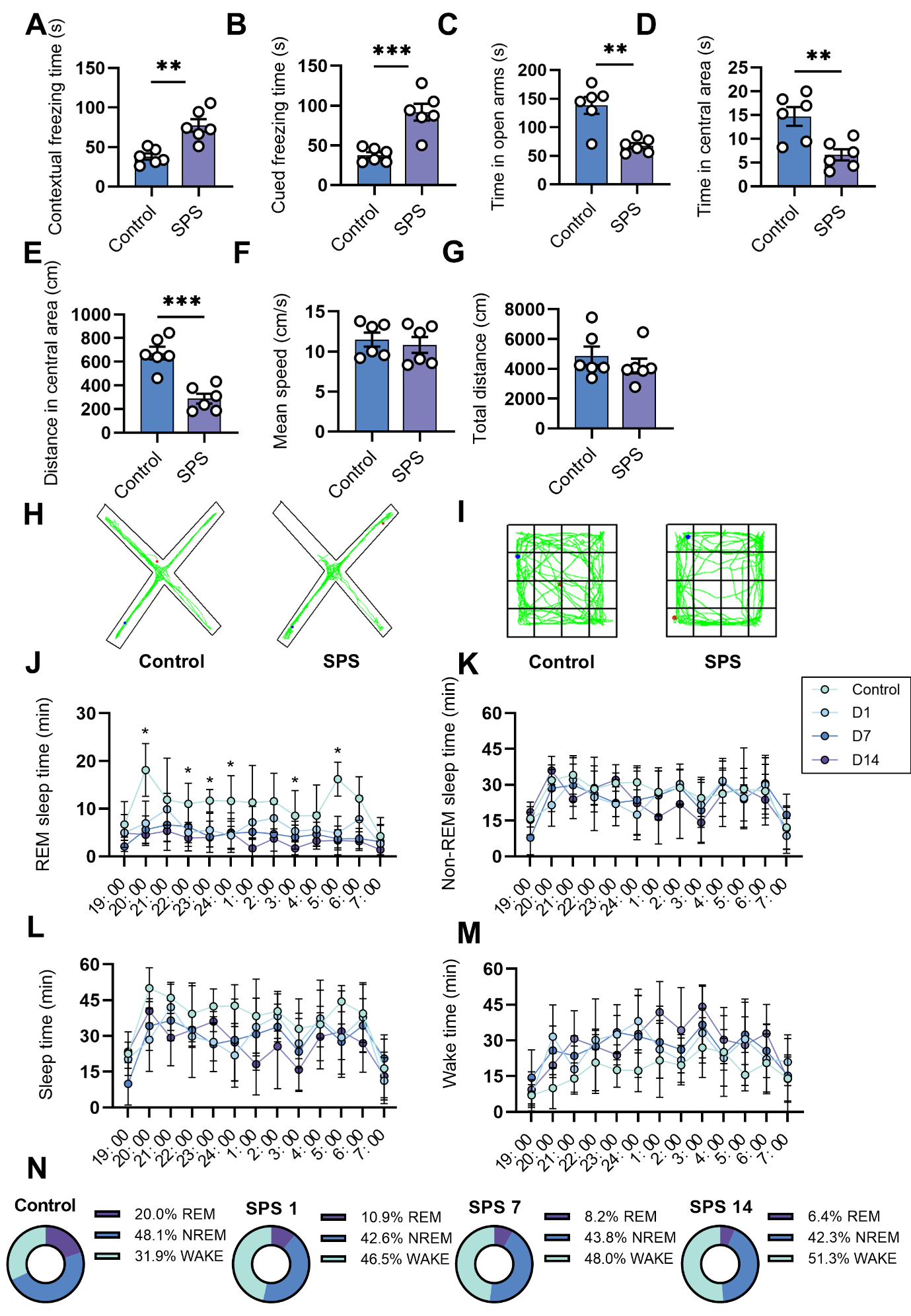
**

**Suppl Fig.1 SPS induces PTSD-like behavior and sleep disturbances in mice.** (A) Freezing time of contextual fear memory (Independent student *t*-test). (B) Freezing time of cued fear memory (Independent student *t*-test). (C) Time spent exploring the open arm in the elevated plus maze test (Independent student *t*-test). (D) Time spent in the center area in the open field test (Independent student *t*-test). (E) Distance traveled in the central area in the open field test (Independent student *t*-test). (F) Total distance in the open field test (Independent student *t*-test). (G) Mean speed in the open field test (Independent student *t*-test). (H) Representative traces in the elevated plus maze test (Independent student *t*-test). (I) Representative traces in the open field test (Independent student *t*-test). (J-M) Hourly REM, NREM, Sleep, WAKE time duration in mice during the 19:00-7:00 monitoring time range (Two-way ANOVA). (N) Pie chart of sleep chronology in mice. Data are expressed as mean ± *SEM*. **p* < 0.05, ***p* < 0.01, and ****p* < 0.001 between groups.


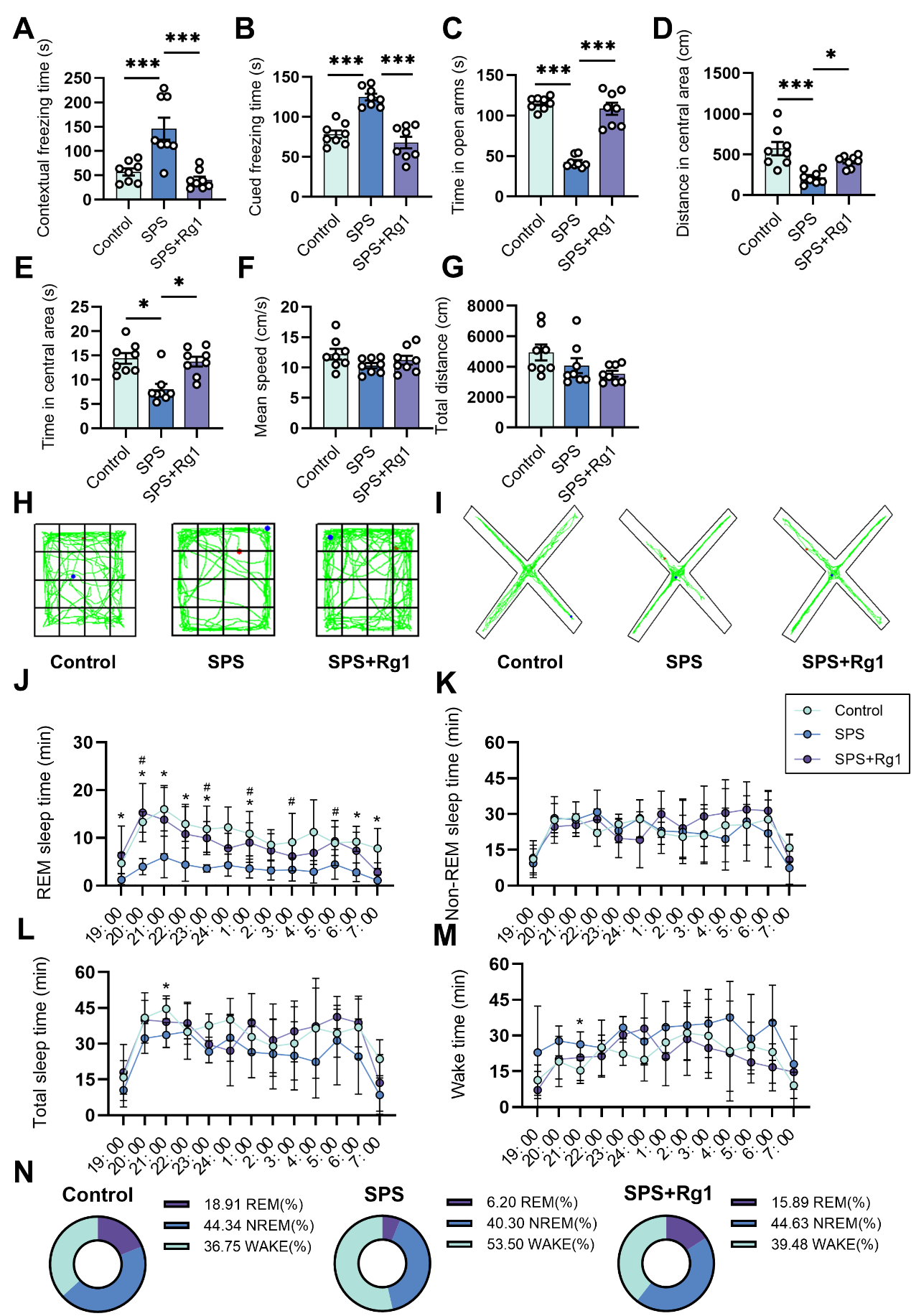


**Suppl Fig.2 Ginsenoside Rg1 prevented PTSD-like behavior and sleep disturbances in SPS mice.** (A) Freezing time of contextual fear memory (one-way ANOVA followed by Tukey-Kramer test). (B) Freezing time of cued fear memory (one-way ANOVA followed by Tukey-Kramer test). (C) Time spent exploring the open arm in the elevated plus maze test (one-way ANOVA followed by Tukey-Kramer test). (D) Distance traveled in the central area in the open field test (one-way ANOVA followed by Tukey-Kramer test). (E) Time spent in the center area in the open field test (one-way ANOVA followed by Tukey-Kramer test). (F) Mean speed in the open field test (one-way ANOVA followed by Tukey-Kramer test). (G) Total distance in the open field test (one-way ANOVA followed by Tukey-Kramer test). (H) Representative traces in the open field test (one-way ANOVA followed by Tukey-Kramer test). (I) Representative traces in the elevated plus maze test (one-way ANOVA followed by Tukey-Kramer test). (J-M) Hourly REM, NREM, Sleep, WAKE time duration in mice during the 19:00-7:00 monitoring time range (Two-way ANOVA). (N) Pie chart of sleep chronology in mice. Data are expressed as mean ± *SEM*. **p* < 0.05, ***p* < 0.01, and ****p* < 0.001 between groups.
